# Ultrasound Evaluation for Shortening the Door-to-Puncture Time During Endovascular Treatment of Intracranial Vessel Occlusion

**DOI:** 10.7759/cureus.83093

**Published:** 2025-04-27

**Authors:** Shinya Yamaguchi, Masato Osaki, Mio Yokoi, Mariya Hokazono, Taisuke Kitamura, Kayo Wakisaka, Tetsuro Sayama, Shuji Arakawa, Shigeru Fujimoto, Koji Yoshimoto

**Affiliations:** 1 Department of Neurosurgery, Steel Memorial Yawata Hospital, Kitakyushu, JPN; 2 Department of Cerebrovascular Disease, Steel Memorial Yawata Hospital, Kitakyushu, JPN; 3 Department of Medicine and Clinical Science, Graduate School of Medical Sciences, Kyushu University, Fukuoka, JPN; 4 Division of Neurology, Department of Medicine, Jichi Medical University, Tochigi, JPN; 5 Department of Neurosurgery, Graduate School of Medical Sciences, Kyushu University, Fukuoka, JPN

**Keywords:** common carotid artery (cca), door-to-puncture time, endovascular treatment (evt), large vessel occlusion (lvo), ultrasound evaluation

## Abstract

Objective: Concerning endovascular treatment for acute ischemic stroke with intracranial vessel occlusion, shortening the door-to-puncture time (DTP) improves the patient’s outcome. To determine endovascular treatment, magnetic resonance angiography or computed tomography angiography is performed for occluded vessel detection. Another detection method of internal carotid artery (ICA) occlusion or middle cerebral artery first segment (M1) occlusion is ultrasound (US). Bilateral flow pattern analysis of common carotid arteries by US leads to the diagnosis of ICA or M1 occlusion within a few minutes. Moreover, it can be conducted in the emergency department. The addition of the US for the initial evaluation of vessel occlusion can shorten the DTP. In this study, we evaluated the effectiveness of carotid artery US imaging in detecting large vessel occlusion (LVO) and shortening the DTP.

Materials and methods: This is a retrospective case-control study. Our analysis was based on the data from 150 patients with LVO or medium vessel occlusion who underwent endovascular revascularization treatment at our hospital between January 2015 and December 2022. Among them, 104 patients who had an anterior circulation vessel occlusion were included. They were divided into the US evaluation group and the non-US evaluation group, and their characteristics, treatment time course, and outcomes were compared.

Results: This study included 104 patients with a median age of 81 years (interquartile range: 73-89 years), 57.7% were females, and the pre-stroke modified Rankin Scale (mRS) median was 0.5 (interquartile range: 0-3). Our cohort included advanced aged patients; therefore, this study included 56.7% of patients over 80 years old and 35.6% of pre-stroke mRS over 3. The US (US group) and non-US (non-US group) evaluation groups included 54 and 50 patients, respectively. As magnetic resonance imaging evaluation in the non-US group was performed over the 4.5 hours delayed arrival of patients from the last known well (LKW) to consider the evaluation of tPA administration, selection bias occurred. The US group included high National Institutes of Health Stroke Scale (NIHSS) patients (P = 0.0152) and more ICA occlusions (P = 0.0146). Onset (LKW) to door time was shorter in the US group (median, 75 min (35-146.5 minutes)) than the non-US group (median, 179 minutes (47.3-432.8 minutes); P = 0.0426), and the DTP was shorter for the US group (median, 75.5 minutes (63.8-87.3 minutes)) than for the non-US group (median, 85 minutes (67-129 minutes); P = 0.0102). Statistical difference was not seen in puncture to reperfusion time among the US group (median, 71.5 minutes (51-114 minutes)) and non-US group (median, 67 minutes (42.3-98.5 minutes); P = 0.5581). The onset (LKW) to reperfusion was shorter for the US group (median, 251 minutes (201-327.3 minutes)) than for the non-US group (median, 319 minutes (200-633.5 minutes); P = 0.0348). No statistical differences were seen for thrombolysis in cerebral infarction grade 2b-3 after treatment, improvement of NIHSS, and mRS at 90 days.

Conclusion: US is a useful imaging method to identify an anterior circulation LVO. It can distinguish patients with severe internal carotid or middle cerebral artery occlusion from medium vessel occlusion or other peripheral vessel occlusions. US helps to shorten the DTP time of LVO.

## Introduction

Acute ischemic stroke owing to large vessel occlusion (LVO) requires vessel recanalization as soon as feasible for favorable clinical outcomes [1-3]. Therefore, detection of LVO is critical. Magnetic resonance angiography (MRA) or three-dimensional computed tomography angiography (CTA) are usually used for LVO detection [4-6]. Magnetic resonance imaging (MRI) and MRA are effective modalities to evaluate the infarction area and detect the occluded vessel. However, as these imaging methods require transfer of patients from the emergency department to the MRI room and the MRI is a long procedure, these steps are time-consuming. Moreover, MRI scans are easily degraded by the patient’s body motion. CTA is an effective modality to detect occluded vessels and evaluate the cerebral flow by perfusion imaging. However, CTA also involves the transfer of patients from the emergency department to the computed tomography (CT) room, and CTA also takes a considerable amount of time. In addition, CTA needs contrast media, which sometimes burdens patients with chronic kidney disease. Carotid artery ultrasound (US) imaging, conventionally used to evaluate carotid artery plaque and stenosis, provides a simpler method to determine the flow pattern of the carotid artery. Using US imaging, an intracranial LVO can be detected from the flow pattern analysis of the bilateral common carotid artery (CCA). An end-diastolic (ED) velocity of 0 cm/sec (dampened pattern) at the LVO side of the CCA indicates internal carotid artery (ICA) occlusion [7,8]. An ED ratio is calculated by dividing the ED flow velocity on the non-occluded side by that on the occluded side, with an elevated ED ratio ≥1.3 indicating an occlusion of the distal ICA or middle cerebral artery first segment (M1) [7]. Yasaka et al. reported that the accuracy of ICA occlusion diagnosis by ED ratio elevation to >4.0 was 97% and that the accuracy of M1 occlusion diagnosis by ED ratio elevation from 1.3 to 4.0 was 93% [7]. US can evaluate CCA flow for a few minutes, can be conducted in the emergency department, and does not need contrast media. The addition of US during the initial evaluation of LVO suspected patients have the possibility to shorten the DTP. Our aim in this study was to evaluate the effectiveness of using carotid artery US imaging to detect LVO, estimate the door-to-puncture (DTP) time, and assess the recanalization treatment outcome.

## Materials and methods

Patient selection　

This is a retrospective case-control study. Between January 2015 and December 2022, we performed revascularization treatment in 150 patients with acute ischemic stroke in our institution. The inclusion criteria were as follows: 1) The patient has symptoms of acute ischemic stroke and no intracranial hemorrhage on initial imaging; 2) age ≥18 years; 3) Alberta Stroke Program Early CT Score (ASPECTS) ≥6; and 4) arterial occlusion of the ICA, M1, middle cerebral artery second segment (M2), or other peripheral vessel is diagnosed on US or MRA or hyperdense vessel on non-contrast CT. Exclusion criteria were as follows: 1) vessel occlusion with posterior circulation; 2) transferred patients who were diagnosed with occluded vessel by imaging at a previous hospital; 3) patients who could not eat by themselves; and 4) patients who had other reasons that the principal investigators judged inappropriate. All study participants provided written informed consent, and the study design was approved by the Steel Memorial Yawata Hospital Institutional Review Board (No. 21-62-02). The diagnosis and treatment for acute ischemic stroke were conducted in compliance with the guidelines of the American Heart Association/American Stroke Association and Japan Stroke Society.

Clinical and imaging evaluation before treatment

**Figure 1:**
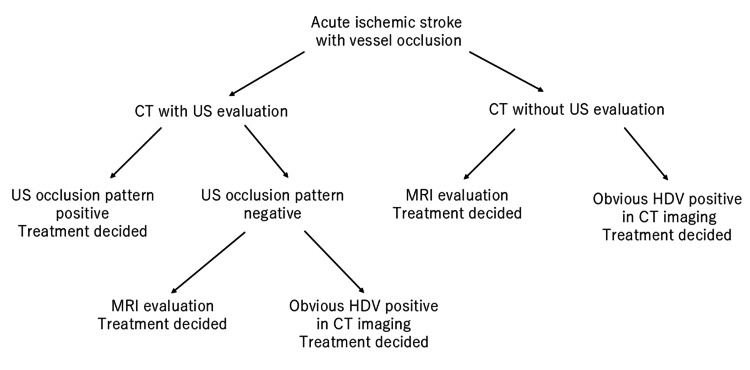
Flow chart shows evaluation methods for treatment decision. Abbreviation: CT, computed tomography; HDV, hyperdense vessel; MRI, magnetic resonance imaging; US, ultrasound

The primary evaluations of patients were performed by a vascular neurologist or neurosurgeon. Pre-treatment neurological evaluations were performed according to the National Institutes of Health Stroke Scale (NIHSS). Direct-arrival and in-hospital patients were diagnosed with infarction based on their clinical symptoms and non-contrast CT imaging. In the absence of any contraindication for thrombolytic therapy, tissue plasminogen activator (tPA) was administered at 0.6 mg per kg body weight. After non-contrast CT, LVOs were basically detected by US or MRA. If the first contact stroke team included a doctor who was able to detect LVO by US imaging, the US evaluation was performed (US group), and if the first contact stroke team did not include a doctor who was able to detect LVO by US, an MRI evaluation was performed (non-US group). Among the doctors who conducted the initial examination for acute ischemic stroke, half of them could be examined by US. We randomly divided half of the month for US diagnosis and half of the month for non-US diagnosis, day by day. Neurologists who use US imaging for daily examinations of carotid artery stenosis took charge of the US evaluation, which was performed concurrently with physical examination, treatment preparation, and tPA administration. If US imaging did not reveal the occlusion pattern, MRA evaluation was considered. In the non-US evaluation group, magnetic resonance imaging (MRI) and MRA were performed after non-contrast CT imaging. Patients who arrived at the hospital with 4.5 hours delay from the time last known well (LKW) were evaluated using MRI and MRA to assess any mismatch between the clinical manifestations and extent of the lesion on diffusion-weighted imaging (DWI) and fluid-attenuated inversion recovery (FLAIR) scans, and an indication to administer tPA was considered. To shorten the MRI evaluation time, imaging methods were restricted to DWI, FLAIR, and intracranial and cervical MRA (about nine minutes). Among the no-occlusion pattern patients in the US group and the patients in the non-US group, magnetic resonance imaging (MRI) and MRA were performed after non-contrast CT imaging. If obvious hyperdense vessel (HDV) signs matching clinical symptoms were detected by non-contrast CT, recanalization treatment was performed without MRI evaluation [9,10]. A non-contrast CT scan was taken with a 2-mm slice thickness with multiplanar reconstruction, and an HDV on CT was referred to as an occluded vessel [9,10]. However, HDV in CT imaging is sometimes difficult to judge as positive. Thus, the doctor in charge was entrusted to judge whether to skip the MRI. The flowchart on treatment decisions is shown in Figure 1.

Ultrasound evaluation

All carotid US examinations were performed by vascular neurologists using the VIVID S6 system (GE Healthcare Japan, Hino, Tokyo, Japan). A linear US probe was used, with a central frequency of 7.5 MHz for both the imaging and pulsed Doppler transducers. Throughout the examination, the angle of the Doppler transducer to the CCA was maintained at 60°. Blood flow was measured at the distal side of the observable CCA. Observation of a dampened CCA flow (ED velocity of 0 cm/s) on the affected side was indicative of an occlusion of the main trunk of the ICA [7,8]. An elevation of the ED ratio (CCA ED velocity on the non-occlusion side divided by the velocity on the side of occlusion) ≥1.3 is considered an LVO of the distal ICA to the main trunk of the MCA [7]. Confirmation of these findings was an indication to proceed with revascularization therapy for the treatment of an acute ischemic stroke with LVO.

Treatment and post-treatment clinical evaluation

The recanalization treatments were performed between January 2015 and December 2018 using a stent retriever alone [4-6] or a Direct Aspiration First Pass Technique (ADAPT) [11] through a 9Fr balloon tip guiding catheter. A combined technique, using a combination of an aspiration catheter and a stent retriever through a 9Fr balloon tip guiding catheter, was mainly used from January 2019 to December 2022 [12-14]. Remote aspiration thrombectomy with a balloon-inflated guiding catheter [15], intra-arterial injection of urokinase [16], percutaneous transluminal angioplasty, and carotid artery stenting were used as needed. The angiographic result was evaluated using the Thrombolysis in Cerebral Infarction (TICI) grading system [17]. Post-neurological evaluation was performed using the NIHSS score at post-treatment days 0, 1, 3, 7, and 21, as well as the day of discharge. An improvement of more than four NIHSS points after treatment was deemed significant. Intracranial hemorrhage (ICH) with deterioration by more than four NIHSS points after treatment was judged as symptomatic ICH. External decompression was performed for malignant cerebral edema. A modified Rankin scale (mRS) score of ≤2 at 90 days was evaluated. The population in the area of our hospital is relatively senior, and patients often have age-related disabilities before LVO. These patients did not reach an mRS score of 0-2, even if the revascularization treatment was effective. Taking this into consideration, we used NIHSS improvement (≥4 points) after treatment and mRS score of ≤3 at 90 days as references.

Statistical analysis

Statistical analyses were performed using JMP (version 14.0, SAS Institute, Cary, NC, USA). Inter-group differences in the proportions of characteristics such as female sex, transport situation, hypertension, diabetes, dyslipidemia, chronic kidney disease, atrial fibrillation, pre-stroke mRS≥3, HDV positive in CT imaging, MRI evaluation, intravenous tPA, left side of occlusion, occluded vessels at treatment, TICI 2b-3, and NIHSS improvement were analyzed using the Pearson’s chi-squared test. Comparison of variables such as age, pre-stroke mRS, ASPECTS, NIHSS before treatment, onset (LKW) to door, DTP time, puncture to reperfusion, onset (LKW) to reperfusion, mRS ≤2 at 90 days, and mRS ≤3 at 90 days were performed using the Wilcoxon’s rank sum test. Symptomatic intracranial hemorrhage (SICH) and external decompression were analyzed using Fisher’s exact test. Effects were considered statistically significant if P < 0.05.

## Results

**Figure 2:**
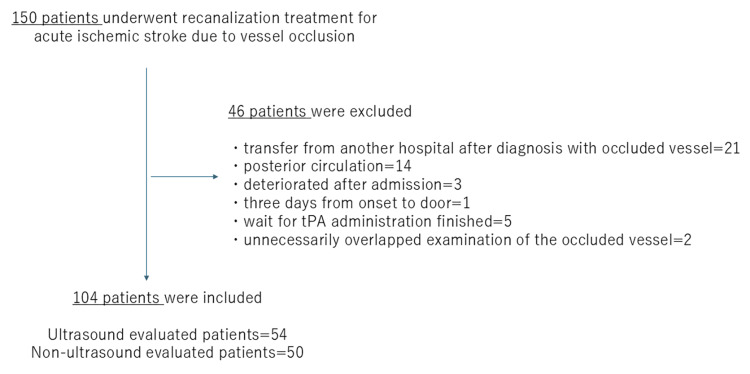
Participant flow diagram illustrating reasons for inclusion and exclusion. Abbreviation: tPA, tissue plasminogen activator

We included 104 of 150 patients for analysis in this study. The flow diagram illustrates the reasons for patient exclusion (Figure 2). The study group of 104 patients included 60 women and had a median age of 81 years (interquartile range: 73-89 years). As a feature of the demographics of our area, the study population had advanced age: 56.7% (59/104) of the treated patients were over the age of 80 years, and 35.6% (37/104) had a pre-infarction mRS score of ≥3 (Table 1). The 104 patients were divided between the US (n = 54) and non-US (n = 50) evaluation groups.

**Table 1:** Age and pre-stroke mRS of the included patients with acute ischemic stroke in our cohort Abbreviations: mRS, modified Rankin scale.

Variables	All patients (n = 104)
Age (years)	
≤59	1 (1.0%)
60–69	12 (11.5%)
70–79	32 (30.8%)
80–89	36 (34.6%)
≥90	23 (22.1%)
Pre-stroke mRS*	
0–2	67 (64.4%)
3–5	37 (35.6%)

In this study, when the occlusion pattern of the US indicated internal carotid artery occlusion (ICO) or middle cerebral artery first segment occlusion (M1O), the sensitivity and specificity of the US were 90.7% and 81.8%, respectively. Regarding sensitivity, four cases of M1O were not diagnosed by US. Regarding specificity, two cases in the US group showed an occlusion pattern. However, during treatment, the occlusion vessel was the second segment of the middle cerebral artery. Tissue-plasminogen activator (tPA) was administered in these two cases. We think that the decline in specificity is influenced by the tPA. Reports on the sensitivity and specificity of CTA or MRA for acute ischemic stroke could not be gathered. However, these have been predicted to be near 100%, as limited to the range of the depicted vessel by CTA and MRA. This is a disadvantage of the US evaluation.

The baseline characteristics of both patient groups are shown in Table 2. The non-US group included more in-hospital patients (P = 0.0290). The US group patients had more atrial fibrillation complications than the non-US group (P = 0.0304). In the non-US group, occluded vessels were more frequently evaluated by MRI (70.0%: P < 0.0001) than in the US group. The proportion of treatment decisions by HDV in CT showed no significant difference between the two groups. TPA was more frequently administered in the US group (P < 0.0001) because the non-US group included patients with unknown onset stroke who were transferred with a 4.5-hour delay from the LKW time. The NIHSS scores before treatment were significantly higher in the US group (P = 0.0152). ICA occlusions were more prevalent in the US group (P = 0.0146), which likely explains the high NIHSS scores in these patients. Due to these factors, the patients in the US group have a possibility of having a worse outcome than those in the non-US group.

**Table 2:** Baseline characteristics of the included patients with acute ischemic stroke in our cohort *Values are presented as median (interquartile range). †Statistically significant. Abbreviations: ASPECTS, Alberta Stroke Program Early CT Score; CT, computed tomography; HDV, hyperdense vessel; ICA, internal carotid artery; MRI, magnetic resonance imaging; mRS, modified Rankin scale; M1, middle cerebral artery first segment; M2, middle cerebral artery second segment; NIHSS, National Institutes of Health Stroke Scale; tPA, tissue plasminogen activator; Tx, therapy; US, ultrasound.

Variable	US group (n = 54)	non-US group (n = 50)	Test statistic	P-value
Age (years)*	81 (75.6–89)	81.5 (70.8–89)	0.6005	0.4384
Female (%)	51.9	64.0	1.570	0.2013
Transport situation (%)			4.767	0.0290^†^
Direct	88.9	72.0		
In-hospital	11.1	28.0		
Hypertension (%)	79.6	70.0	1.284	0.2572
Diabetes (%)	37.0	28.0	0.964	0.3263
Dyslipidemia (%)	44.4	40.0	0.210	0.6467
Chronic kidney disease (%)	61.1	48.0	1.802	0.1795
Atrial fibrillation (%)	77.8	58.0	4.688	0.0304^†^
Pre-stroke mRS*	0 (0–3)	1 (0–3)	0.0000	1.0000
Pre-stroke mRS ≧3 (%)	37.0	34.0	0.104	0.7465
ASPECTS*	10 (8–10)	10 (8–10)	0.5784	0.4469
MRI evaluation (%)	9.3	70.0	40.469	<0.0001^†^
Tx decision by HDV in CT imaging (%)	18.5	30.0	1.874	0.1710
Intravenous tPA (%)	74.1	34.0	16.833	<0.0001^†^
NIHSS before Tx*	20 (16–23)	16.5 (9–22)	5.9093	0.0152^†^
Left side of occlusion (%)	57,4	56.0	0.021	0.8894
Occluded vessel at Tx (%)			8.458	0.0146^†^
ICA	51.9	26.0		
M1	31.5	38.0		
M2 or other peripheral vessels	16.7	36.0		

Time course data and treatment results are shown in Table 3. Onset (LKW) to door time was shorter in the US group (median, 75 minutes (35-146.5 minutes)) than non-US group (median, 179 minutes (47.3-432.8 minutes); P = 0.0426), and the DTP was shorter for the US group (median, 75.5 minutes (63.8-87.3 minutes)) than for the non-US group (median, 85 minutes (67-129 minutes); P = 0.0102). Statistical difference was not seen in puncture to reperfusion time among the US group (median, 71.5 minutes (51-114 minutes)) and non-US group (median, 67 minutes (42.3-98.5 minutes); P = 0.5581). The onset (LKW) to reperfusion was shorter for the US group (median, 251 minutes (201-327.3 min)) than for the non-US group (median, 319 minutes (200-633.5 minutes); P = 0.0348). Even though the US group comprised more patients with high NIHSS scores and proximal vessel occlusion who were more likely to develop a large infarction area, there were no statistically significant differences in the angiographic outcome, symptomatic ICH, external decompression, NIHSS improvement, and mRS score at 90 days between the two groups. 

**Table 3:** Time course data and treatment results in a cohort of 104 patients with acute ischemic stroke. *Values are presented as median (interquartile range). †Statistically significant. Abbreviations: DTP, door-to-puncture; LKW, last known well; mRS, modified Rankin scale; NIHSS, National Institutes of Health Stroke Scale; SICH, symptomatic intracranial hemorrhage; TICI, thrombolysis in cerebral infarction grade; US, ultrasound.

Variable	US group (n = 54)	non-US group (n = 50)	Test statistics	P-value
Onset (LKW) to door (min)*	75 (35–146.5)	179 (47.3–432.8)	4.1128	0.0426^†^
DTP time (min)*	75.5 (63.8–87.3)	85 (67–129)	6.6080	0.0102^†^
Puncture to reperfusion (min)*	71.5 (51–114)	67 (42.3–98.5)	0.3429	0.5581
Onset (LKW) to reperfusion(min)*	251 (201–327.3)	319 (200–633.5)	4.4574	0.0348^†^
TICI 2b-3 (%)	88.9	80.0	1.576	0.2094
SICH (%)	1.99	0	-	1.0000
External decompression (%)	0	2	-	0.4808
NIHSS improvement (%)	74.7	68.0	0.467	0.4945
mRS ≤2 at 90days (%)	14.8	24.0	1.410	0.2350
mRS ≤3 at 90days (%)	40.8	46.0	0.293	0.5886

Representative case

**Figure 3:**
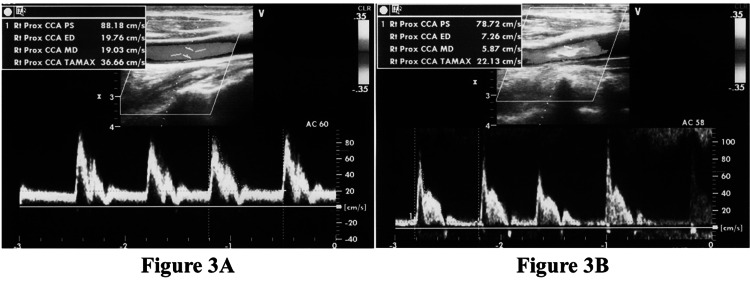
Ultrasound evaluation of a 73-year-old man with left hemiparesis. A: CCA waveform for the left carotid artery without a suspected occlusion. The ED velocity is 19.76 cm/s. B: CCA waveform for the right carotid artery with a suspected occlusion. Compared to the left side, the ED velocity is decreased to 7.26 cm/s. The calculated ED ratio is 2.72, and occlusion of right ICA or MCA is suspected. Abbreviations: CCA, common carotid artery; ED, end-diastolic; ICA, internal carotid artery; MCA, middle cerebral artery.

A 73-year-old man was transferred to our hospital for a possible endovascular treatment. The period between his LKW time to the time of his arrival at our hospital was 230 minutes. The patient presented with left hemiparesis and facial palsy, with a conjugate deviation to the right. His NIHSS score was 16, and his ASPECT score was 6. On the US evaluation, the ED flow velocity of the left CCA was 19.76 cm/s (Figure 3A). The ED flow velocity of the right CCA decreased to 7.26 cm/s (Figure 3B). The ED ratio was 2.72, indicative of the right ICA or MCA occlusion. Endovascular treatment for right distal ICA occlusion was performed with a DTP time of 66 minutes, and complete TICI 3 recanalization was achieved. After rehabilitation, the patient had slight upper limb paresis but could walk without a crutch, and his mRS score was 3 at 90 days.

## Discussion

Many previous reports showed that a delay from onset to recanalization led to a negative outcome [1-3]. A short DTP time was reported to be associated with a positive outcome [18-20]. In this study, we focused on DTP time because it can be shortened by changing the evaluation method or optimizing the effort of medical teams. Shorter target DTP times had been proposed previously, namely, 75 minutes in the HERMES report in 2016 [3] and 60 minutes in the report by McTaggart et al. in 2017 [21]. Regardless of the proposed ideal DTP time estimates, the aim should always be to save time from presentation to recanalization. However, these ideal times are often difficult to achieve because of limitations in manpower or institutional evaluation systems required for LVO detection. Several methods were reported as methods to shorten door to needle or DTP [19,20,22,23]. These include direct transfer to angiosuite [20], mobile stroke unit with CTA [23], and to review workflow and shorten each step to treatment [19, 22]. All these attempts aim to shorten the time from onset to recanalization. Similar to previous reports, our facility reviews the workflow of each case and feedback from the conference, which includes the doctors, radiology technicians, and nurses for the purpose of shortening treatment time and improving outcomes [19,22]. However, each facility restricts the available methods. Therefore, each institution must establish a suitable evaluation system. In our study, 18.5% (10/54) of the US group patients achieved a DTP time of <60 minutes, whereas 50.0% (27/54) achieved a DTP time of <75 minutes. The median DTP time of the US group was 75.5 minutes, which was shorter than the 90-minute period reported in the SWIFT PRIME study in which LVO was detected by CTA and MRA [5] and the 113-minute period in the EXTENDED-IA study in which LVO was detected by CTA [6]. Evaluation of LVO by US contributed to DTP time shortening. Considering that every four minutes of delay from emergency department arrival to reperfusion worsened disability outcome in one patient per 100 [3], we think that the shorter DTP time will have the possibility to improve the prognosis of the US group patients with LVO and severe stroke symptoms.

Several factors were considered as the reason for a short DTP in the US group. Non-US group patients were more frequently evaluated by MRI, which took approximately nine minutes. In this study, MRI methods were restricted to DWI, FLAIR, and intracranial and cervical MRA. Nevertheless, the MRI evaluation time was longer than the US evaluation time. The US evaluation time was a few minutes in this study. MRI evaluation was performed in 9.3% of the US group and in 70.0% of the non-US group. Skipping the MRI would be a significant factor in the short DTP of the US group. As another reason, all US evaluations were performed concurrently with examination and preparation for the treatment in the emergency room. In contrast, MRI evaluation includes the time taken to transfer the patient to the MRI suite. These factors may have lengthened the DTP time in the non-US group. Comparing CTA and US, the advantages of US include that this evaluation can be conducted in the emergency room without contrast media. The patients in our cohort included much advanced ages, and 54.8% of them had chronic kidney disease. It is advantageous that US does not need contrast media in such cases. Another demerit of our study is a lack of perfusion. However, in this study, the influence without perfusion was little. Inclusion criteria of ASPECT≥6 in this study did not increase symptomatic ICH or external decompression. Mobile stroke units with CTA are reported to shorten DTP [23]. As mobile stroke units did not expand adequately in the Japanese countryside, it is difficult to compare. We think that if mobile stroke units could perform US evaluation, it would have the possibility to shorten DTP without contrast media. Probably the best evaluation method is different in each facility. Considering the best of our facility, the US evaluation led to shortening DTP and decline the patient’s burden due to no contrast media.

Among the 150 patients included in this study, 21 were transferred from another hospital for a thrombectomy after being diagnosed with vessel occlusion. The occlusion vessels of these patients were diagnosed by MRI (10 had an IC occlusion, 8 had an M1 occlusion, and 3 had an M2 segment occlusion) at the previous hospital before transfer. These transferred patients received the treatment after clinical and neurological evaluation, blood sampling, explanation of the endovascular treatment, insertion of a urinary catheter, and insertion of an intravenous drip root. US evaluations of the transferred patients were performed randomly in parallel with clinical and neurological evaluation and preparation for endovascular treatment. US evaluation was performed as part of the neurological examination on arrival at our hospital in 12 of these 21 patients (TUS subgroup) and was not performed in 9 patients (TnUS subgroup). The median DTP time for the TUS subgroup was 30.0 (range, 25.0-40.8) minutes, and that for the TnUS subgroup was 40.0 (range, 27.5-55.5) minutes. The DTP time was similar in the TUS and TnUS subgroups (P = 0.3737). We could not point out a clear reason for the delay within the TnUS group. However, we considered that this result might indicate that the influence of the US evaluation on the DTP time was little when performed concurrently with examination and preparation for the endovascular treatment.

A particular advantage of US evaluation is that it can shorten the DTP time in patients with proximal vessel occlusion and high NIHSS scores. In these patients, recanalization must be performed as soon as possible, because their condition is prone to deteriorate. Another merit of US evaluation is that it can be performed at the bedside concurrently with physical and neurological examinations, which saves time. On the other hand, the disadvantages of US evaluation include the requirement for staff to be familiar with this technique and the inability to detect peripheral occlusion. In our study, 13 of the 54 cases in the US group showed no occlusion pattern. Occluded lesions in these 13 cases included six MCA branch occlusions, four M1 occlusions, one anterior cerebral artery (ACA) occlusion, one MCA and ACA branch occlusion, and one IC stenosis. DTP times of these 13 cases (median 77 minutes; interquartile range, 65.5-108.5) were not statistically different from those in the non-US group (P = 0.3286). We think that the delay in US group patients with no-occlusion pattern is short because the evaluation time of US is short and performed in the emergency room concurrently with examination and preparation. It is a demerit of US evaluation not to detect patients with peripheral branch occlusion. However, it is a great merit to detect LVO and to quicken treatment initiation in LVO patients with high NIHSS scores. Similarly to our method of using US for vessel occlusion detection, Ishibashi et al. diagnosed LVO by the ED ratio elevated above 1.4 [24]. They compared DTP time in 23 patients diagnosed with LVO by US evaluation, and six cases who were misdiagnosed by the initial US evaluation and had MRA added to the diagnostic work-up. The authors showed short DTP time for patients imaged by using US only. The method of comparison was different from ours; the US evaluation can shorten door-to-puncture time.

In this study, infarction areas were evaluated by non-contrast CT [25], and we referred to the HDVs on non-contrast CT to estimate the occluded vessels [9,10]. We performed CT evaluation with a 2 mm slice thickness and multiplanar reconstruction (coronal and sagittal) for HDV detection. The percentage of occluded vessels detected as HDVs was 53.6% in our study. The HDV signs were associated with occlusion in 62-67% of previously reported cases [9,10]. We showed the percentage of treatment decisions by HDV in CT in Table 2. There was no significant difference between the two groups. The treatment decision by HDV in CT has the possibility to lead to short DTP. However, in this study, the proportion of treatment decisions by HDV in CT was lower in the US group than in the non-US group. Therefore, we think that the treatment decision by HDV in CT in the US group has a lower impact on shortening DTP time than that in the non-US group. In this study, 46.3% of patients in the US group showed no HDV signs on CT scans. When the occlusion flow pattern is detected by US imaging, a decision to perform recanalization treatment can be made even if the patients do not show HDV signs on CT.

In this series, we were able to shorten the DTP time by using US evaluation, but the proportion of mRS scores of 0-2 at 90 days was poor compared to that in previous reports [4-6]. The reason for the poor prognosis is that our series included many old patients with higher pre-stroke mRS scores. In fact, despite neurological improvement after recanalization of their occluded vessel, the condition of some patients deteriorated owing to other physical conditions, such as heart failure, pneumonia or carcinoma. Furthermore, we found no differences in the improvement of prognosis between the US and non-US groups. This could be because patients in the US group were mainly treated for proximal occlusion, whereas non-US group patients were mostly treated for peripheral occlusion. In this study, the ASPECT≥6 was an inclusion criterion. The patients with over 4.5 hours delay from the last known well were evaluated by MRI for tPA administration. Some patients in the non-US group with severe ICO and M1O may be excluded due to the low ASPECTS. As a result, the ICO and severe stroke patients were more in the US group. Considering these differences, we propose that US evaluation may improve the outcome by shortening the DTP time in patients with proximal vessel occlusion, which could easily lead to large infarct lesions. This may lead to a prognosis similar to that of the non-US group patients with peripheral occlusions.

Apart from a shorter DTP time, US imaging has other advantages: it is non-invasive, can be used at the bedside, visualizes the vessel wall as well as the intravessel lesion, provides information about blood flow, can be repeated as necessary without complications, and can be performed concurrently with tPA therapy. In our study, occlusion flow patterns were improved after endovascular treatment. Thus, flow pattern evaluation by US imaging was effective in monitoring the continuity of recanalization.

Limitations

This study has several limitations. First, this study involved a relatively small group of patients. Second, physicians who are familiar with US evaluation were needed in the stroke team. Third, the US group was treated for proximal vessel occlusion, whereas the non-US group was treated for peripheral vessel occlusion and had tPA administered less frequently. Fourth, the mRS scores at 90 days were not good. There are many patients at advanced age in Japan, which is a reflection of this country's real-world data. These people have a low mRS score before the stroke episode. Moreover, because our facility's covered area is a progressively aging region, our cohort included a greater age group with other comorbidities. In addition, more severe NIHSS patients with ICO were included in the US group from the selection bias. Such characteristics of both groups complicate the generalization and interpretation of study results. For these reasons, our results limited the generalizability of the findings. But the effect of shortening DTP for improvement has possibility to be underestimated. We would reexamine to support the validity of our observations in future studies, after changing the study design and with a larger sample size.

## Conclusions

The use of carotid US evaluation for LVO detection in patients presenting with acute ischemic stroke shortened DTP times. US imaging has the possibility to help to identify patients with proximal LVO, such as IC or M1 occlusion, and to shorten the DTP time of proximal LVO patients without delay for medium vessel or other peripheral vessel occlusions. Identification of dampened pattern of CCA flow or an elevated ED ratio by US imaging should be considered as an indication for thrombectomy. The shortened DTP achieved with US evaluation may improve patient outcomes.
